# A Self-Compassion and Mindfulness-Based Cognitive Mobile Intervention (Serene) for Depression, Anxiety, and Stress: Promoting Adaptive Emotional Regulation and Wisdom

**DOI:** 10.3389/fpsyg.2021.648087

**Published:** 2021-03-22

**Authors:** Mohamed Al-Refae, Amr Al-Refae, Melanie Munroe, Nicole A. Sardella, Michel Ferrari

**Affiliations:** The Wisdom and Identity Lab, Department of Applied Psychology and Human Development, University of Toronto, Toronto, ON, Canada

**Keywords:** mindfulness, self-compassion, serene, depression, wisdom, emotion regulation, app, CBT

## Abstract

**Introduction:** Many individuals and families are currently experiencing a high level of COVID-19-related stress and are struggling to find helpful coping mechanisms. Mindfulness-based interventions are becoming an increasingly popular treatment for individuals experiencing depression and chronic levels of stress. The app (Serene) draws from scholarly evidence on the efficacy of mindfulness meditations and builds on the pre-existing apps by incorporating techniques that are used in some therapies such as cognitive behavioral therapy and mindfulness-based cognitive therapy.

**Methods:** Participants were randomly assigned to a 4-week mindfulness and self-compassion-based cognitive smartphone intervention (Serene) or a wait-list control group. They were instructed to engage in self-compassion and mindfulness practices and a cognitive restructuring task. They also completed measures that evaluated their levels of depression, stress, anxiety, self-compassion, wisdom, psychological well-being, and subjective well-being. The intervention group was also instructed to track their weekly engagement with the app. Standardized effect sizes for between-group differences were calculated using Cohen's *d* for complete case analyses.

**Results:** Complete case analyses from baseline to the end of this randomized controlled trial demonstrated significant moderate between-group differences for depressive symptoms (*d* = −0.43) and decisiveness (*d* = 0.34). Moderate between-group differences were also found for self-compassion (*d* = 0.6) such that significant improvements in self-kindness, common humanity, mindfulness and decreases in self-judgement, isolation, and overidentification were observed. A small between-group difference was found for emotional regulation (*d* = 0.28). Moreover, a significant moderate within-group decrease in stress (*d* = −0.52) and anxiety symptoms (*d* = −0.47) was also observed in the intervention group.

**Conclusions:** Serene is an effective intervention that promotes increased levels of self-compassion and emotional regulation. Engaging with Serene may help reduce depressive symptoms through mindfulness, self-compassion, and cognitive restructuring which help reduce overidentification with one's negative emotions. As individuals rebalance their thinking through cognitive restructuring, they can identify the varying stressors in their life, develop action plans and engage in adaptive coping strategies to address them. Serene may promote greater self-understanding which may provide one with a more balanced perspective on their current upsetting situations to positively transform their challenges during the pandemic.

## Introduction

In today's world, individuals are struggling to find new ways to cope with the COVID-19 pandemic. Compared with other adversities, COVID-19 has introduced new challenges that have led to greater shifts in societal and psychological functioning. For instance, social distancing measures have created a void and feelings of isolation that have exacerbated pre-existing subclinical symptoms of depression, anxiety, and chronic stress among the general population (Dawel et al., 2020; Galea et al., 2020). Relative to other one-sided adversities, the pandemic has resulted in a global challenge that we all face together. In addition to the physiological impact this pandemic has created, it has also resulted in an underlying rise in mental health challenges. With the rise in pharmacological interventions to tackle these issues, many researchers have begun to also explore psychosocial measures that can reduce one's distress. Finding new ways to effectively manage one's stressors during the pandemic can help alleviate psychological symptoms and improve one's overall well-being.

Due to the ongoing pandemic, a need for technologically assisted interventions has increased. Multiple interventions that promote self-compassion show considerable promise for the treatment of stress, anxiety, and depression. One recent meta-analysis looked at multiple self-compassion-based interventions on various outcome measures and found evidence for significant increases in self-compassion, mindfulness, and well-being and decreases in depression, anxiety, and psychological distress (Kirby et al., 2017). Wahbeh et al. (2014) also found individuals significantly preferred internet-delivered mindfulness interventions over in-person group or individual mindfulness meditation interventions. Therefore, in the face of the ongoing challenges of the pandemic, a tool that incorporates these practices may supersede previous practices and strategies that individuals had relied on but can no longer engage in (e.g., in-person, group-based support).

Self-compassion is considered the antithesis of some of the consequences of stress, anxiety, and depression. It is a multidimensional construct that includes self-kindness (having greater self-understanding and kindness to oneself in moments of distress), common humanity (a perception of one's experiences as being part of the larger human condition), and mindfulness (a balanced and present awareness of one's thoughts, feelings, and body sensations while not overidentifying with them). Through self-compassion, individuals can achieve greater understanding of their experiences and suffering and can therefore be more willing to move beyond them (Neff, 2003a). For instance, Neff (2003a) believes self-compassion can reduce feelings of isolation and promote the engagement in adaptive coping mechanisms. One meta-analysis by MacBeth and Gumley (2012) found self-compassion can significantly decrease symptoms of stress, anxiety, and depression. Self-compassion can also act as a protective factor against psychosocial stress, which is influenced by how individuals cognitively appraise their stressors (Breines et al., 2015; Bluth et al., 2016). Neff et al. (2007) also found self-compassion may act as a protective factor against feelings of anxiety that arise in the face of a stressor, even while accounting for differing levels of self-esteem. In the face of the pandemic, self-compassion may be a critical ingredient in dealing with the stress, panic, and resulting depression one may experience (Zeller et al., 2015). Linardon (2020) suggests self-compassion and mindfulness may be promoted through cost-effective, mobile-based technological means and that they may offer relief from psychological distress. Therefore, a smartphone-mobile app that offers an avenue to develop a self-compassionate stance may help improve clinical outcomes.

Allen and Leary (2010) also explain how self-compassion can afford individuals the ability to deal with stressors effectively through cognitive reappraisal, an adaptive form of emotional regulation. Adaptive emotion regulation strategies allow individuals to cope with their unwanted negative emotions without suppressing or ignoring them (Bridges et al., 2004; Grawe, 2007; Glück, 2021). Cognitive reappraisal (a process conducted in cognitive restructuring) is the ability to reframe a stressful or upsetting situation in a more positive manner by having an open attitude while engaging in positive thinking. Self-compassion, in of itself, results in the ability to not overidentify with one's negative emotions (a component of this cognitive process). Multiple studies have tested this notion and found a role for self-compassion in reducing one's focus on negative emotions and thoughts (Neff et al., 2005; Leary et al., 2007). Leary et al. (2007) additionally found individuals can take responsibility for the negative circumstances that have occurred while not overidentifying with the negative affect that is experienced. Many current available apps do not employ explicit cognitive behavioral strategies. A review by Wasil et al. (2019) on the current inclusion of evidence-based therapeutic measures for depression and anxiety has demonstrated a lack of these components (cognitive restructuring and problem-solving mechanisms) in current mental health apps.

Neff et al. (2007) also found self-compassion may reduce self-criticism and rumination, two factors that play a role in maintaining depressive and anxiety symptoms. Zhang et al. (2019) similarly found self-compassion mediated the relationship between self-criticism and depressive symptoms. A recent study by Xu et al. (2020) also examined the role of cognitive reappraisal in predicting the relationship between perceived stress and anxiety symptoms in individuals who were isolated during COVID-19. They found this emotion regulation strategy significantly moderated the relationship between perceived stress and anxiety symptoms. Another study by Diedrich et al. (2016) found participants who engaged in self-compassion practices prior to the use of explicit cognitive restructuring had greater improvements in depression. Fortuna and Vallejo (2015) explain how engaging in mindfulness and self-compassionate practices may facilitate the cognitive restructuring process in the context of traumatic and upsetting situations. The authors also describe how both mindfulness and cognitive restructuring, in the context of mindfulness-based cognitive therapy (MBCT), together foster resiliency in the face of adversity to promote faster recovery (Fortuna and Vallejo, 2015). Therefore, the integration of self-compassionate techniques with explicit cognitive restructuring may provide additive benefits toward improving anxiety and depressive symptoms and promoting resilience in the face of stressors.

Wisdom, a closely related construct is also construed as a multidimensional trait. It includes the ability to make appropriate social decisions in complex situations, exhibiting greater impulse control and emotional regulation, and demonstrating prosocial behaviors such as compassion and empathy. Wisdom also encompasses the ability to see reality clearly which entails increased self-awareness and deep insight into one's mind and self (Ardelt, 2003; Bangen et al., 2013). Practicing self-compassion also helps promote wisdom. Neff argues that self-compassion includes forgiveness, loving, and acceptance of the self despite flaws which can be construed as a form of “emotional [or affective] wisdom” (Neff, 2003b; Germer and Siegel, 2012). Studies have demonstrated the link between self-compassion and reflective and affective wisdom (Ardelt, 2003; Neff et al., 2007). Previous studies have also found an association between self-compassion and wisdom and their link to improved well-being and greater life satisfaction (Ardelt, 1997, 2000; Neff, 2003b; Ardelt and Jeste, 2018). Glück et al. (2019) also found wiser individuals presented with higher levels of openness, reflectivity, empathy, and emotional regulation. Therefore, wiser individuals may be able to deal with the current pandemic's stressors and other pre-existing challenges more effectively.

The purpose of this study was to test the efficacy of a wellness smartphone application (Serene) on measures of stress, depression, anxiety, self-compassion, well-being, and wisdom. Specifically, this study examined whether various mindfulness meditations and a cognitive restructuring task (which requires the development of an action plan and incorporation of emotion-focused and problem-focused coping mechanisms) will influence these outcomes (see Lazarus and Folkman, 1984 for more on coping mechanisms). We hypothesized there would be a direct improvement on the well-being, stress, depression, and anxiety measures as well as an increase in self-compassion and wisdom scores.

## Materials and Methods

### Participants

Participants who were 18 years old and above were eligible to participate in the study. Participants were required to be residents of Canada and to own an iPhone with iOS 13+. No exclusions based on diagnosis with any disorder was used. No exclusions based on current engagement with any mindfulness practices or any other treatments were also used.

Table 1 demonstrates the sample's characteristics for the complete case analyses. Participants ranged in age from 18-66 years (Mean Age = 25.24, SD = 8.74). Most participants were female in the intervention group (80.8%) and waitlist control (WL) condition (77.0%). Most individuals in both conditions also self-identified as White (43.6% in Serene, 31.0% in WL), followed by individuals identifying as East and South Asian. Most participants were current university students (35.9% in Serene and 41% in WL) or had completed an undergraduate degree (28.2% in Serene and 33.3% in WL). The current employment status varied: 32.1% of participants in Serene and 42.5% of participants in the WL were unemployed students. 23.1% of participants in Serene and 20.7% of participants in the WL were employed full-time. Among the current sample, only a few participants were unemployed non-students (4.8%).

**Table 1:** Demographic comparisons between serene and waitlist control (complete case analysis).

	**Whole sample** **(*n* = 165)**	**Serene** **(*n* = 78)**	**Waitlist control** **(*n* = 87)**	**Statistics**
**Age**				
Mean (SD)	25.24 (8.74)	26.13 (8.42)	24.46 (8.99)	*t*_(147)_ = 1.17, *p* = 0.25
Age range	18-66	18-54	18-66	
Did not disclose	(*n* = 16)	(*n* = 8)	(*n* = 8)	
**Ethnicity (*****N*****, %)**				*X^2^*(*N* = 165, df = 10) = 28.87, *p* = 0.001
Indigenous	1 (0.6%)	1 (1.3%)	0 (0%)	
White	61 (37.0%)	34 (43.6%)	27 (31.0%)	
South Asian	25 (15.2%)	5 (6.4%)	20 (23.0%)	
East Asian	34 (20.6%)	19 (24.4%)	15 (17.2%)	
South East Asian	4 (2.4%)	2 (2.6%)	2 (2.3%)	
Filipino	9 (5.5%)	2 (2.6%)	7 (8.0%)	
Latin American/Hispanic	3 (1.8%)	0 (0%)	3 (3.4%)	
West Indian	1 (0.6%)	1 (1.3%)	0 (0%)	
Black	3 (1.8%)	0 (0%)	3 (3.4%)	
Arab/West Asian	9 (5.5%)	3 (3.8%)	6 (6.9%)	
Other	15 (9.1%)	11 (14.1%)	4 (4.6%)	
**Gender (*****N*****, %)**				*X^2^*(*N* = 164, df = 2) = 4.31, *p* = 0.12
Male	32 (19.4%)	12 (15.4%)	20 (23.0%)	
Female	130 (78.8%)	63 (80.8%)	67 (77.0%)	
Gender non-conforming	2 (1.2%)	2 (2.6%)	0 (0%)	
Did not disclose	1 (0.6%)	1 (1.3%)	0 (0%)	
**Highest level of education (*N*, %)**				*X^2^*(*N* = 165, df = 4) = 2.25, *p* = 0.69
Grade school	0 (0%)	0 (0%)	0 (0%)	
High school diploma or GED	19 (11.5%)	11 (14.1%)	8 (9.2%)	
College or trade School	13 (7.9%)	7 (9.0%)	6 (6.9%)	
Some University	64 (38.8%)	28 (35.9%)	36 (41.4%)	
University undergraduate degree	51 (30.9%)	22 (28.2%)	29 (33.3%)	
Post graduate degree	18 (10.9%)	10 (12.8%)	8 (9.2%)	
**Current employment status (*****N*****, %)**				*X^2^*(*N* = 165, df = 6) = 12.56, *p* = 0.051
Employed full-time	36 (21.8%)	18 (23.1%)	18 (20.7%)	
Employed part-time	16 (9.7%)	13 (16.7%)	3 (3.4%)	
Unemployed	8 (4.8%)	4 (5.1%)	4 (4.6%)	
Student employed part-time or full-time	36 (21.8%)	15 (19.2%)	21 (24.1%)	
Student not employed	62 (37.6%)	25 (32.1%)	37 (42.5%)	
Retired	2 (1.2%)	0 (0%)	2 (2.3%)	
Homemaker	0 (0%)	0 (0%)	0 (0%)	
Other	5 (3.0%)	3 (3.8%)	2 (2.3%)	
**Diagnosis with mental health disorder (*****N*****, %)**				*X^2^*(*N* = 165, df = 2) = 1.31, *p* = 0.52
Any diagnosis	42 (25.5%)	23 (29.5%)	19 (21.8%)	
No diagnosis	118 (71.5%)	53 (67.9%)	65 (74.7%)	
Did not disclose	5 (3.0%)	2 (2.6%)	3 (3.4%)	
**Diagnosis from whole sample (*****N*****, %)**				*X^2^*(*N* = 165, df = 1)
Generalized anxiety disorder (GAD)	23 (13.9%)	11 (14.1%)	12 (13.8%)	0.003, *p* = 0.95
Depression	21 (12.7%)	13 (16.7%)	8 (9.2%)	2.07, *p* = 0.15
Bipolar disorder	1 (0.6%)	0 (0%)	1 (1.1%)	1.29, *p* = 0.26
Panic disorder	6 (3.6%)	4 (5.1%)	2 (2.3%)	1.29, *p* = 0.26
Obsessive compulsive disorder (OCD)	6 (3.6%)	4 (5.1%)	2 (2.3%)	0.95, *p* = 0.33
Social anxiety disorder	5 (3.0%)	4 (5.1%)	1 (1.1%)	2.34, *p* = 0.13
Eating disorder	2 (1.2%)	2 (2.6%)	0 (0%)	3.02, *p* = 0.08
Posttraumatic stress disorder	4 (2.4%)	2 (2.6%)	2 (2.3%)	3.02, *p* = 0.08
Did not disclose	6 (3.6%)	2 (2.6%)	4 (4.6%)	0.012, *p* = 0.91
Other	17 (10.3%)	10 (12.8%)	7 (8.0%)	
**Medication Use for MHDs from total sample (*****N*****, %)**				*X^2^*(*N* = 165, df = 3) = 3.49, *p* = 0.32
Taking medication	16 (9.7%)	9 (11.5%)	7 (8.0%)	
Not taking medication	129 (78.2%)	61 (78.2%)	68 (78.2%)	
Did not disclose	4 (2.4%)	3 (3.8%)	1 (1.1%)	
N/A	16 (9.7%)	5 (6.4%)	11 (12.6%)	
**Other Treatments for MHDs (e.g., Therapy)–(*****N*****, %)**				*X^2^*(*N* = 165, df = 3) = 0.14, *p* = 0.99
Receiving treatment	24 (14.5%)	12 (15.4%)	12 (13.8%)	
Not receiving treatment	121 (73.3%)	57 (73.1%)	64 (73.6%)	
Did not disclose	2 (1.2%)	1 (1.3%)	1 (1.1%)	
N/A	18 (10.9%)	8 (10.3%)	10 (11.5%)	
**Knowledge/practice of mindfulness/meditations (*****N*****, %)**				*X^2^*(*N* = 163, df = 3) = 2.03, *p* = 0.36
Regular practitioner/Great Knowledge	12 (7.3%)	8 (10.3%)	4 (4.6%)	
Dabbled with some practices/Know a little on mindfulness	114 (69.1%)	54 (69.2%)	60 (69.0%)	
Never practiced/don't know anything on mindfulness	37 (22.4%)	16 (20.5%)	21 (24.1%)	
Did not disclose	2 (1.2%)	0 (0%)	2 (2.3%)	
**Currently Engaging in mindfulness practice (*****N*****, %)**				*X^2^*(*N* = 162, df = 3) = 4.25, *p* = 0.04
Yes	49 (29.7%)	29 (37.2%)	20 (23.0%)	
No	113 (68.5%)	47 (60.3%)	66 (75.9%)	
Did not disclose	3 (1.8%)	2 (2.6%)	1 (1.1%)	

Almost 25% of the sample had a diagnosis with a mental health disorder. The majority were diagnosed with generalized anxiety disorder (14.1% in Serene, 13.8% in WL) and depression (16.7% in Serene, 9.2% in WL). Only 9.7% of the whole sample (38.0% of those who had any diagnosis) were taking medications for their mental health disorders while 14.5% were receiving treatment for their mental health disorder from the whole sample (57.14% from those who self-identified with any diagnosis). These medications included stimulants, antidepressants (selective serotonin reuptake inhibitors and serotonin-norepinephrine reuptake inhibitors), benzodiazepines, and antipsychotics. Treatments used included traditional face-to-face cognitive behavioral therapy (CBT), internet-delivered CBT, psychoanalytic psychotherapy, family counseling, individual counseling, and other forms of therapy.

Only 7.3% of the sample were regular practitioners or had great knowledge of mindfulness or other forms of meditation at the beginning of the study. 22.4% had never practiced or heard of mindfulness or meditations while about 69% had engaged in some mindfulness or meditative practice or had knowledge about it. Moreover, 37.2% of participants in the intervention group and 23.0% of participants in the waitlist control condition were engaging in these practices at the beginning of the study.

### Procedure

This randomized waitlist-controlled study underwent approval by the Research Ethics Board at the University of Toronto. The study was carried out from August 29, 2020 to December 6, 2020. Participants were recruited through online Facebook groups, Kijiji, Reddit groups targeted for Canadians and email listservs at the University of Toronto from August 29 to November 7. Participants who met criteria for participation and who consented to participate in the study were provided with a link to complete the first survey. After completion of the survey, participants were provided with Canadian (national, local, and university-based) mental health resources to be used if they encountered any upsetting feelings or emotions. They completed a demographics questionnaire that included questions pertaining to their age, gender, employment status, ethnicity, education levels, income, and current diagnoses with mental health disorders.

**Figure 1:**
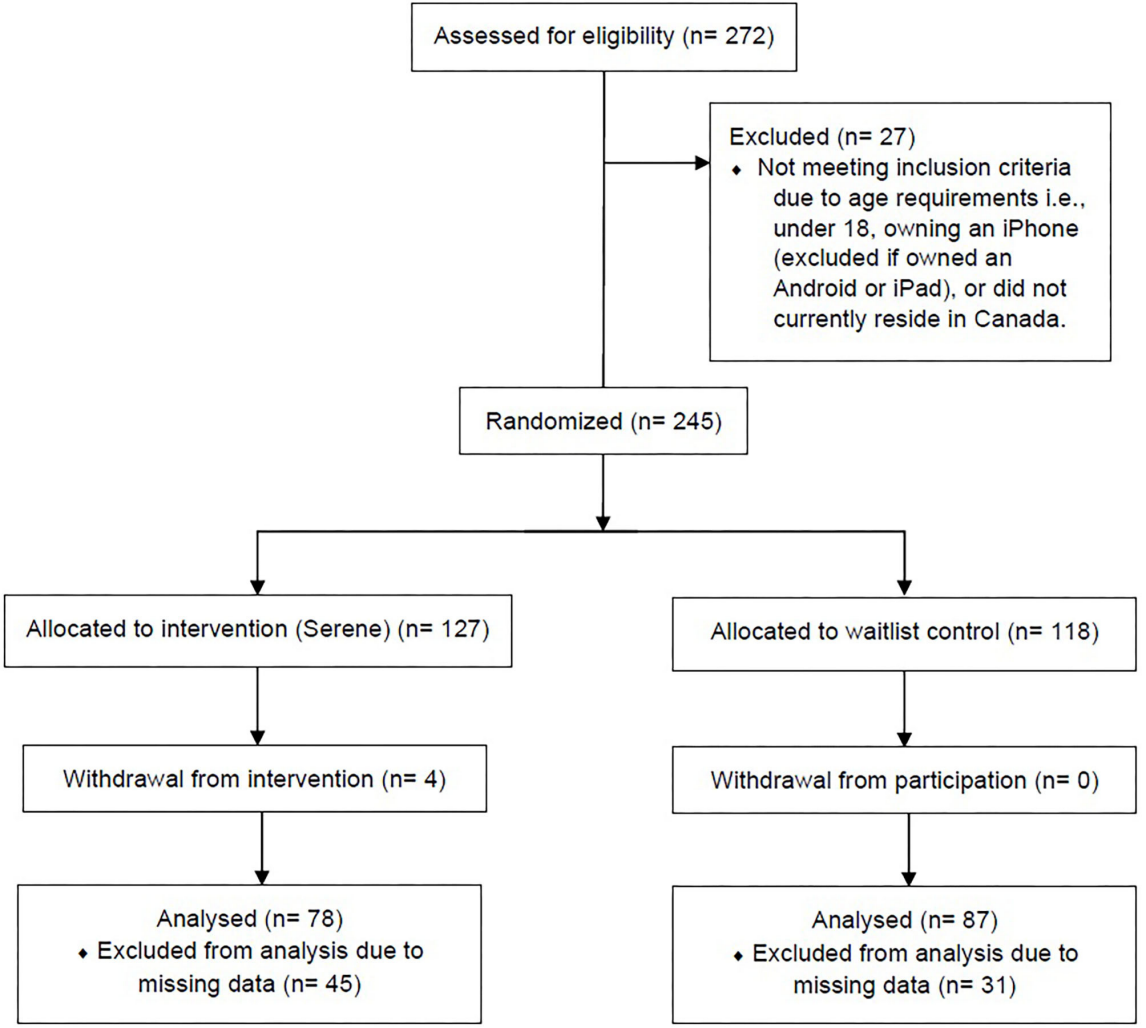
CONSORT study flow diagram.

After the completion of the first survey, participants were randomized to the intervention group (Serene app) or a waitlist control group. Participants were randomized into their respective group using a generated list from randomizer.org^1^. A description of the participant flow diagram is displayed in Figure 1. Following completion of the first survey, a research team member allocated participants to their group. Due to the nature of the study, participants were unblinded to their group allocation. Both groups of participants filled out a final survey using self-report measures for depression, anxiety, stress, self-compassion, well-being, and wisdom. Questions pertaining to engagement with the app (e.g., average mindfulness meditations used per week) were asked. Moreover, participants were asked questions pertaining to their subjective well-being at the end of the study. Participants in the waitlist control condition were instructed that they would receive the app following completion of their final survey (after 30 days). They continued with any current treatments or mindfulness practices they were using. Participants in the intervention group were asked to download the TestFlight app, used for testing beta apps. Through TestFlight, participants were able to download the Serene app. They were instructed to do at least one mindfulness meditation a day of their choice. They were also asked to do a cognitive restructuring task, as needed. No minimum or maximum amount of cognitive restructuring tasks were required. Depending on each participant, they may choose to perform one task (i.e., they may continue to modify one plan to address one situation) or engage in more than one task throughout the study. A journaling section was available for use but was not required for participants to engage in. At the end of the study, participants in the intervention group were offered the app for lifetime use. Participants in the waitlist control group also received the app following completion of their final surveys.

### Intervention

The app offered a psychoeducation component (See Supplementary Material 1) that detailed the benefits of mindfulness and self-compassion practices. A section on dealing with unexpected distress or upsetting feelings that may arise as one becomes more aware of their current state (called backdraft) was also included (Germer and Neff, 2013). Moreover, a section on cognitive restructuring, its benefits, and examples for each step in this process was also included. Some benefits and examples of healthy coping strategies that may be incorporated into one's action plan were also provided. Moreover, some techniques for mindful journaling and self-compassionate writing were also included.

Mindfulness meditations (both formal and informal) varied in length and content. Mindfulness is a psychological process that involves attention to the present moment while acknowledging and accepting one's emotions and thoughts through a non-judgemental and open fashion. Focusing on the present is thought to increase awareness to how one's thoughts are connected to specific adaptive or maladaptive behaviors. This may then lead to greater self-insight, self-acceptance, and self-compassion (Brown et al., 2007). Available mindfulness meditations included breathing meditations, mindful walking, sleep-focused meditations, awareness-based meditations, gratitude meditations, meditations focused on reducing stress and anxiety, body scan meditations and self-compassion-based meditations (including loving-kindness meditations). Nature sounds were also used as an informal/unguided meditative method. Many of these methods align with trauma-informed mindfulness practices. According to Treleaven (2018) and Crews et al. (2016), focusing one's attention on the breath and internal experience may trigger trauma-related symptoms or emotional distress that may be uncomfortable for the individual. Therefore, the music/nature sound section was included to be used as a way for users to focus on the sounds as opposed to the breath. Many of the included mindfulness meditations also included a waterfall sound in the background. Moreover, mindful meditations that shift the focus on physical activity or awareness to one's scenery as opposed to the breath (e.g., mindful walking) were also included as they have been proven to be as effective as mindfulness meditations that focus on the breath (Taylor et al., 2020).

The cognitive restructuring task was adapted from two evidence-based therapies, cognitive behavioral therapy and mindfulness-based cognitive therapy. Cognitive restructuring involves the identification and modification of unhelpful thoughts or beliefs underlying one's feelings and developing and applying actions plans to modify one's behaviors. Combining it with mindfulness and self-compassion practices promotes greater awareness of the processes that maintain these habitual patterns of unhelpful thoughts and beliefs. Developing greater awareness helps individuals observe their distressing situation from a more balanced perspective, thereby allowing them to reframe these unhelpful thoughts or feelings more effectively (Segal et al., 2013; Fortuna and Vallejo, 2015). Step 1 of this task involved identifying a situation or an unwanted thought that may be upsetting or uncomfortable. Step 2 involved identifying the associated strongest feeling with the situation. Step 3 involved identifying the thought that is most strongly related to the feeling and situation (e.g., sadness/depression or guilt/shame). Examples were provided for each of these feelings as well. Step 4 involved identifying evidence for and against the thought patterns. Step 5 involved evaluating the evidence to identify whether there was sufficient, partial or no evidence to these thoughts. Based on the selected evaluation, individuals were guided to engage in various next steps. If an individual found no evidence for the thought, they were invited to find alternative ways of thinking or looking at the situation. They were also invited to form an action plan to cope with any emotional distress that may arise from the situation or unwanted thoughts. If there was partial or full support for the thought, individuals were also invited to form an action plan on how to deal with their upsetting emotions or unwanted thoughts. They were also invited to develop a plan to address the situation (e.g., engaging in a healthy coping mechanism or planning steps to directly address the situation). The app also provided a brief card with these questions with a breathing exercise to be used informally. This feature was only unlocked once individuals engaged in at least 1 formal cognitive restructuring task.

In the journaling section, individuals were prompted to rate their current mood using an Emoji scale. They were invited to record their current progress (e.g., their progress on an action plan) or to note down any current thoughts or feelings about their current state.

### Measures

#### Depression, Anxiety, and Stress

The Depression, Anxiety, and Stress Scale (DASS-21, Lovibond and Lovibond, 1995b) is a 21-item measure used to assess levels of stress, anxiety and depression on a seven-point Likert scale. It is divided into three subscales that measure: depression (e.g., “I felt that I had nothing to look forward to”), anxiety (e.g., “I was worried about situations in which I might panic and make a fool of myself”), and stress (e.g., “I found myself getting agitated”). Total scores for each subscale are calculated by computing a mean of all items on each subscale and multiplying the score by 2. Antony et al. (1998), Henry and Crawford (2005) and Lovibond and Lovibond (1995a) reported an acceptable internal consistency reliability (α = 0.82–0.97) for this scale in both clinical and non-clinical samples. For the current sample the internal reliability was acceptable at baseline for the stress subscale (α = 0.80), anxiety subscale (α = 0.81), and depression subscale (α = 0.87). The internal reliability was also acceptable at the end of the study for the stress subscale (α = 0.88), anxiety subscale (α = 0.86), and depression subscale (α = 0.92).

#### Wisdom

The San-Diego Wisdom scale (SD-WISE; Thomas et al., 2019) is a 24-item measure used to assess levels of wisdom based on its neurobiological model on a five-point Likert scale. It is further divided into six subscales that measure: social advising (e.g., “I am good at perceiving how others are feeling”), decisiveness (e.g., “I have trouble making decisions”—reverse scored), emotional regulation (e.g., “I am able to recover well from emotional stress”), insight (e.g., “ I take time to reflect on my thoughts”), prosocial behavior (e.g., “I treat others the way I would like to be treated”), and tolerance for divergent values (e.g., “ I enjoy learning things about other cultures”). Total wisdom scores and subscale scores are calculated by summing the scores of the items. Thomas et al. (2019) found that the internal reliability (α = 0.72) for this scale is acceptable. For the current sample the internal reliability was acceptable at baseline for the total wisdom score (α = 0.80), social advising subscale (α = 0.67), decisiveness subscale (α = 0.80), emotional regulation subscale (α = 0.69), insight subscale (α = 0.74) and tolerance for divergent values subscale (α = 0.66). For the prosocial behavior subscale, Cronbach's alpha was 0.45. The internal reliability was also acceptable at the end of the study for the total wisdom score (α = 0.81), social advising subscale (α = 0.67), decisiveness subscale (α = 0.80), emotional regulation subscale (α = 0.69), insight subscale (α = 0.77), and tolerance for divergent values subscale (α = 0.61). For the prosocial behavior subscale, Cronbach's alpha was 0.48.

The 12-item Abbreviated Three-Dimensional Wisdom scale (3D-WS-12; Thomas et al., 2017) is a measure that is also used to assess levels of wisdom on a five-point Likert scale. Items on this scale measure levels of affective wisdom (e.g., “Sometimes I feel a real compassion for everyone”—reverse scored), reflective wisdom (e.g., “Sometimes I get so charged up emotionally that I am unable to consider many ways of dealing with my problems”) and cognitive wisdom (e.g., “I try to anticipate and avoid situations where there is a likely chance I will have to think in depth about something”). Total scores are calculated by computing a mean of all items on the scale. Thomas et al. (2017) found that the internal reliability for this scale (α = 0.73) is acceptable. For the current sample, the internal reliability was acceptable at baseline for this scale (α = 0.73). The internal reliability was also acceptable at the end of the study for this scale (α = 0.65).

#### Psychological Well-Being

The Psychological Well-being scale (Ryff and Keyes, 1995) is an 18-item measure used to assess levels of psychological well-being on a seven-point Likert scale. It is further divided into six subscales that measure: personal growth (e.g., “For me, life has been a continuous process of learning, changing, and growth), purpose in life (e.g., “Some people wander aimlessly through life, but I am not one of them”) positive relations with others (e.g., “Maintaining close relationships has been difficult and frustrating for me”—reverse scored), self-acceptance (e.g., “When I look at the story of my life, I am pleased with how things have turned out so far”), environmental mastery (e.g., “In general, I feel I am in charge of the situation in which I live”), and autonomy (e.g., “I have confidence in my own opinions, even if they are different from the way most other people think”). Subscale scores are calculated by computing a sum of each of the items on their respective subscales. Ryff and Keyes (1995) reported scores between α = 0.33–0.56 for the internal reliability of the six subscales. For the current sample the internal reliability was acceptable at baseline for the autonomy subscale (α = 0.69), environmental mastery subscale (α = 0.60), and self-acceptance subscale (α = 0.68). Cronbach's alpha was 0.59 for the personal growth subscale, 0.57 for the positive relations with others subscale, and 0.50 for the purpose in life subscale. The internal reliability was also acceptable at the end of the study for the autonomy subscale (α = 0.63), environmental mastery subscale (α = 0.62), positive relations with others subscale (α = 0.62), and self-acceptance subscale (α = 0.73). Cronbach's alpha for the personal growth subscale was 0.55 and 0.47 for the purpose in life subscale.

#### Self-Compassion

The Self-Compassion Scale (SCS; Neff, 2003b) is a 26-item measure used to assesses levels of self-compassion on a five-point Likert scale. It is further divided into six subscales that measure: self-kindness (e.g., “I try to be loving toward myself when I'm feeling emotional pain”), self-judgment (e.g., “I can be a bit cold-hearted toward myself when I'm experiencing suffering.”), common humanity (e.g., “When I'm down and out, I remind myself that there are lots of other people in the world feeling like I am”), isolation (e.g., “When I fail at something that's important to me, I tend to feel alone in my failure”), mindfulness (e.g., “When something upsets me I try to keep my emotions in balance”), and over-identified (e.g., “When something upsets me I get carried away with my feelings”). Total self-compassion scores are calculated by reverse scoring the negative subscale items (self-judgement, isolation, over-identified), calculating scores on the positive subscales (self-kindness, common humanity, mindfulness), and then finally calculating the mean of all these subscales. To calculate scores for each subscale, a mean is computed without reverse scoring any of the items. Neff (2003b) found that the internal reliability (α = 0.90) and test–retest consistency (0.93) coefficients for the self-compassion scale are acceptable. The internal reliability for the current sample was acceptable at baseline for the total self-compassion score (α = 0.91), self-kindness subscale (α = 0.84), self-judgment subscale (α = 0.80), common humanity subscale (α = 0.74), isolation subscale (α = 0.75), mindfulness subscale (α = 0.69), and over-identified subscale (α = 0.71). The internal reliability was also acceptable at the end of the study for the total self-compassion score (α = 0.9**2**), self-kindness subscale (α = 0.82), self-judgment subscale (α = 0.87), common humanity subscale (α = 0.80), isolation subscale (α = 0.79), mindfulness subscale (α = 0.68), and over-identified subscale (α = 0.79).

#### Subjective Well-Being

Participants were asked to rate whether engaging in a component of the app helped improve their overall well-being (e.g., use of the mindfulness meditations has helped you improve your overall well-being) by indicating a Yes or No.

#### Engagement

Participants were asked to indicate whether they engaged in mindfulness meditations daily. They were also asked to indicate the amount of mindfulness meditations they engaged in. Participants were also asked to indicate the amount of cognitive restructuring tasks they engaged in (and whether they were new tasks or revisiting an old task), journaling tasks they engaged in, and nature or music sounds they played. Moreover, they were asked to indicate whether they started one of these tasks and ended them and to indicate the reason for doing so.

### Statistical Analysis

Data analysis was performed using SPSS version 27.0. Little's MCAR test was used to analyze patterns of missing data (Little, 1988). The results were consistent with the data being missing completely at random. Due to the size of missing data, we chose to perform a complete case analysis which produces unbiased results compared to the multiple imputation method when the size of missing data is large (Mukaka et al., 2016). The normality and homogeneity of the data were tested and confirmed by the Kolmogorov-Smirnov and Shapiro-wilk tests and by observing the Q-Q plots and considering both skewness of the data and kurtosis. One-way analysis of variance (ANOVA) using Welch's *t*-test was used to compare mean scores on the measures at baseline between the intervention and wait-list control groups. Complete case analysis was performed using General Linear Models (GLM) repeated measures with time x group interactions to assess changes in the intervention and control group from baseline to post-study. Pearson correlations were used to assess correlations within these repeated measures. Together with the pooled standard deviation and mean differences, within and between group effect sizes at post treatment (Cohen's *d*) were computed for the complete case analyses (Cohen, 1977). In the case where normality assumptions were not met, non-parametric Mann–Whitney *U*-tests were used to compare mean differences across the intervention and control group. A non-parametric Friedman test was also used to assesses within-group (intervention) differences where no between-group differences were found for some of the measures. Cohen's *d* was also calculated for these analyses.

## Results

### Engagement With the App (Serene)

Table 2 presents the levels of engagement with the app. On average, participants in the intervention group engaged in 5 meditations a week. Cognitive restructuring was used by 34.8% of the sample in week 1, 17.4–18.4% in weeks 2 and 3, and by 8.7% of participants in the intervention group. Journaling was practiced by 40.6% of participants the intervention group at the end of the first week, followed by 31.9% engagement in week 2 and 21.7–23.2% in the last 2 weeks. On average, nature sounds and the music playlists were used once a week across all weeks.

**Table 2:** Engagement with the app for the intervention group (complete case analysis).

	**Intervention usage for tasks (*n* = 69)**			
	**Week 1**	**Week 2**	**Week 3**	**Week 4**
**Mindfulness meditations**				
Mean (SD)	5.35 (2.08)	4.87 (2.25)	5.04 (4.04)	4.17 (3.21)
**Cognitive restructuring**				
Yes	24 (34.8%)	12 (17.4%)	13 (18.8%)	6 (8.7%)
Mean (SD)	1.42 (0.78)	1.33 (0.89)	1.15 (0.38)	1.00 (0)
No	45 (65.2%)	57 (82.6%)	56 (81.2%)	63 (91.3%)
**Journaling**				
Yes	28 (40.6%)	22 (31.9%)	16 (23.2%)	15 (21.7%)
Mean (SD)	2.32 (2.00)	2.05 (1.81)	3.13 (2.31)	3.27 (2.22)
No	41 (59.4%)	47 (68.1%)	53 (76.8%)	54 (78.3%)
**Nature sounds**				
Mean (SD)	1.18 (1.69)	0.97 (1.66)	0.90 (1.61)	0.70 (1.36)
**Music**				
Mean (SD)	1.66 (2.99)	1.47 (3.49)	0.81 (1.72)	0.88 (1.79)

### Effectiveness of Intervention (Serene)

#### Depression, Anxiety, and Stress

The normality and homogeneity assumptions for stress, anxiety and depression were not met. Therefore, the non-parametric Mann–Whitney *U*-test was used to compare between-group differences in stress, anxiety, and depression from baseline to 4 weeks. No significant baseline differences were found across all three measures using one-way ANOVA. Findings for the depression measure revealed a statistically significant difference between the intervention vs. control group at the end of study using the Mann–Whitney *U*-test, *p* < 0.05, *d* = −0.43, 95% confidence interval [CI: −0.71, –0.16] (Table 3). Findings for the stress measure revealed a significant difference between stress scores in the intervention vs. control group at the end of study using the Mann–Whitney *U*-test, *p* < 0.01. Effect size calculations revealed no statistically significant between-group differences for stress, *d* = −0.25, 95% confidence interval [CI: −0.52, 0.03] (Table 3). A non-parametric Friedman test within the intervention group using Bonferroni correction revealed a significant decrease in stress symptoms, *p* < 0.001, *d*_Repeated Measures_ = −0.52, 95% confidence interval [CI: −0.86, −0.18] at the end of the 4 weeks. Baseline scores indicated a mean level of mild stress levels. Mean levels post-intervention were normal for stress within the intervention group.

**Table 3:** Averages (SD) and effect sizes for serene and waitlist control group (complete case analysis).

**Category**	**Intervention: Serene**	**Waitlist control**	***N***	**Between-group effect size (Int vs. WL)—Cohen's *d***
**Measure**	**Mean (SD)**	**Mean (SD)**		
Depression-pre	16.11 (10.25)	14.75 (10.18)	Int: 69, WL: 75	
Depression-post	10.93 (10.14)	14.1 (11.16)	Int: 69, WL: 75	
Depression change				−0.43
Anxiety-pre	11.36 (8.69)	11.79 (9.36)	Int: 69, WL: 75	
Anxiety-post	8.23 (8.69)	10.0 (9.69)	Int: 69, WL: 75	
Anxiety change				−0.15
Stress-pre	16.52 (8.02)	18.32 (9.75)	Int: 69, WL: 75	
Stress-post	13.04 (9.55)	17.2 (10.54)	Int: 69, WL: 75	
Stress change				−0.25
Self-compassion-pre	2.59 (0.55)	2.72 (0.62)	Int: 78, WL: 87	
Self-compassion-post	2.89 (0.60)	2.67 (0.62)	Int: 78, WL: 87	
Self-compassion change				0.60
Self-kindness-pre	2.63 (0.72)	2.77 (0.76)		
Self-kindness-post	2.83 (0.75)	2.66 (0.76)		
Self-kindness change				0.42
Self-judgement-pre	3.69 (0.67)	3.48 (0.83)		
Self-judgement-post	3.27 (0.83)	3.43 (0.96)		
Self-judgement change				−0.44
Common humanity-pre	2.87 (0.81)	2.93 (0.77)		
Common humanity-post	3.11 (0.78)	2.71 (0.76)		
Common humanity change				0.58
Isolation-pre	3.60 (0.85)	3.43 (0.90)		
Isolation-post	3.20 (0.95)	3.38 (0.95)		
Isolation change				−0.38
Mindfulness-pre	3.07 (0.70)	3.14 (0.69)		
Mindfulness-post	3.23 (0.65)	2.97 (0.71)		
Mindfulness change				0.49
Over-identified-pre	3.69 (0.76)	3.57 (0.88)		
Over-identified-post	3.29 (0.88)	3.50 (0.88)		
Over-identified change				−0.37
Wisdom (3D-WS)-pre	3.20 (0.60)	3.20 (0.55)	Int: 77, WL: 86	
Wisdom (3D-WS)-post	3.28 (0.50)	3.20 (0.48)	Int: 77, WL: 86	
Wisdom (3D-WS) change				0.16
Wisdom (SD-WISE)-pre	85.32 (10.24)	86.31 (9.66)	Int: 77, WL: 86	
Wisdom (SD-WISE)-post	85.88 (9.67)	85.76 (9.85)	Int: 77, WL: 86	
Wisdom (SD-WISE) change				0.11
Emotional regulation-pre	11.44 (3.02)	11.44 (3.00)		
Emotional regulation-post	12.25 (2.76)	11.47 (3.00)		
Emotional regulation change				0.28
Decisiveness-pre	10.49 (3.78)	11.41 (3.67)		
Decisiveness-post	11.53 (3.36)	11.21 (3.75)		
Decisiveness change				0.34
Social advising-pre	14.81 (2.81)	15.28 (2.45)		
Social advising-post	14.87 (2.35)	15.24 (2.66)		
Social advising change				0.04
Insight-pre	15.82 (2.92)	15.92 (2.64)		
Insight-post	15.56 (2.86)	15.80 (2.70)		
Insight change				−0.05
Prosocial behavior-pre	16.19 (2.10)	16.01 (2.34)		
Prosocial behavior-post	15.70 (2.16)	15.63 (2.44)		
Prosocial behavior change				−0.05
Tolerance for divergent values-pre	16.57 (2.45)	16.26 (2.33)		
Tolerance for divergent values-post	15.97 (2.31)	16.42 (2.1)		
Tolerance for divergent values change				−0.33
Personal growth-pre	17.10 (2.98)	17.76 (2.88)	Int: 77, WL: 86	
Personal growth-post	17.43 (3.10)	17.27 (3.05)	Int: 77, WL: 86	
Personal growth change				0.27
Purpose in life-pre	14.16 (3.87)	15.90 (3.72)		
Purpose in life-post	14.48 (3.67)	15.47 (3.65)		
Purpose in life change				0.20
Positive relations with others-pre	14.57 (3.80)	14.37 (3.93)		
Positive relations with others-post	14.38 (4.01)	14.23 (3.82)		
Positive relations with others change				−0.01
Self-acceptance-pre	13.17 (4.07)	13.94 (4.26)		
Self-acceptance-post	13.51 (4.09)	13.98 (4.25)		
Self-acceptance change				0.07
Environmental mastery-pre	13.12 (3.59)	12.78 (3.68)		
Environmental mastery-post	13.01 (3.60)	12.28 (3.94)		
Environmental mastery change				0.11
Autonomy-pre	13.82 (3.93)	14.28 (3.70)		
Autonomy-post	14.22 (3.70)	14.00 (3.33)		
Autonomy change				0.19

The non-parametric Mann–Whitney *U*-test was used to compare between-group differences in anxiety from baseline to 4 weeks. Results revealed no statistically significant differences between anxiety scores in the intervention vs. control group at the end of study, *p* > 0.05, *d* = −0.15, 95% confidence interval [CI: −0.41, 0.11] (Table 3). A non-parametric Friedman test within the intervention group using Bonferroni correction revealed a significant decrease in anxiety symptoms, *p* < 0.001, *d*_Repeated Measures_ = −0.47, 95% confidence interval [CI: −0.95, −0.26] at the end of the 4 weeks. Baseline scores indicated a mean level of moderate anxiety levels. Mean levels post-intervention were mild for anxiety within the intervention group.

#### Self-Compassion

The normality and homogeneity assumptions for the self-compassion total score and subscale measures were met. Therefore, complete case analysis using GLM repeated measures with time x group interactions were conducted for the total self-compassion score and subscale scores. No significant baseline differences were found across these measures using one-way ANOVA. Findings for the total self-compassion score indicated a significant time x group interaction, *p* < 0.001, *d* = 0.60, 95% confidence interval [CI: 0.34, 0.85], such that the Serene group reported significantly greater improvements in self-compassion than the waitlist control group (Tables 3, 4).

**Table 4:** Primary outcomes as a function of group and time of measurement (complete case analysis).

**Outcome of GLMs**	**Time**	**Time X Serene vs. WL**
Self-compassion(T: *n* = 78, WL: *n* = 87)	11.72_1,163_, *p* < 0.001^**^	23.66_1,163_, *p* < 0.001^**^
Self-kindness	0.86_1,163_, *p* = 0.36	8.96_1,163_, *p* = 0.003^**^
Self-judgment	15.82_1,163_, *p* < 0.001^**^	9.54_1,163_, *p* = 0.002^**^
Common humanity	0.50_1,163_, *p* = 0.82	16.82_1,163_, *p* < 0.001^**^
Isolation	11.51_1,163_, *p* < 0.001^**^	7.23_1,163_, *p* = 0.008^**^
Mindfulness	0.04_1,163_, *p* < 0.84	10.50_1,163_, *p* = 0.001^**^
Over-identified	16.89_1,163_, *p* < 0.001^**^	7.79_1,163_, *p* = 0.006^**^
3D-WS(T: *n* = 77, WL: *n* = 86)	1.47_1,161_, *p* = 0.23	1.87_1,161_, *p* = 0.17
Psychological well-being(T: *n* = 77, WL: *n* = 86)		
Positive relations with others	0.61_1,1 61_, *p* = 0.44	0.02_1,161_, *p* = 0.89
Self-acceptance	0.59_1,161_, *p* = 0.45	0.39_1,161_, *p* = 0.54
Environmental mastery	1.84_1,161_, *p* = 0.18	0.79_1,161_, *p* = 0.38
Autonomy	0.78_1,161_, *p* = 0.78	2.38_1,161_, *p* = 0.13
SD-WISE(T: *n* = 77, WL: *n* = 86)	0.0_1,161_, *p* = 0.99	0.98_1,161_, *p* = 0.32
Decisiveness	5.2_1,161_, *p* = 0.024^*^	11.23_1,161_, *p* = 0.001^**^
Emotional regulation	4.81_1,161_, *p* = 0.03^*^	4.28_1,161_, *p* = 0.04^*^

*T, Intervention (Serene App). WL, Waitlist Control. Results are presented as F-values (Fdf1, df2). Exact p-values are also included*.

* *indicates a significance level of p < 0.05*.

** *indicates a significance level of p < 0.001*.

Findings for the positive subscales indicated a significant time x group interaction on self-kindness, *p* = 0.003, *d* = 0.42, 95% confidence interval [CI: 0.14, 0.70], common humanity, *p* < 0.001, *d* = 0.58, 95% confidence interval [CI: 0.30, 0.87], and mindfulness, *p* = 0.001, *d* = 0.49, 95% confidence interval [CI: 0.18, 0.79], such that the Serene group reported significantly greater improvements in self-kindness, common humanity and mindfulness than the waitlist control group (Tables 3, 4). Findings for the negative subscales indicated a significant time x group interaction on self-judgement, *p* = 0.002, *d* = −0.44, 95% confidence interval [CI: −0.74, −0.15], isolation, *p* = 0.008, *d* = −0.38, 95% confidence interval [CI: −0.67, −0.10], and the overidentified subscale, *p* = 0.006, *d* = −0.37, 95% confidence interval [CI: −0.64, −0.10], such that the Serene group reported significantly greater decreases in self-judgment, isolation and overidentification than the waitlist control group (Tables 3, 4).

#### Wisdom

##### 3D-WS-12

The normality and homogeneity assumptions for this measure were met. Therefore, a complete case analysis using GLM repeated measures with a time × group interaction was conducted. No significant baseline differences were found across this measure using one-way ANOVA. Findings for the 3D-WS-12 measure indicated no significant time interaction, *p* = 0.23 and no significant time x group interaction, *p* = 0.17, *d* = 0.16, 95% confidence interval [CI: −0.06, 0.38] (Tables 3, 4).

##### SD-WISE

The normality and homogeneity assumptions for the total score for this measure were met. These assumptions were also met for the decisiveness and emotional regulation subscales only. Therefore, complete case analyses using GLM repeated measures with time × group interactions were conducted for the total wisdom score and the two subscale measures that met these assumptions. No significant baseline differences were found across these measures using one-way ANOVA. Findings for the total wisdom score indicated no significant time x group interaction, *p* = 0.36, *d* = 0.11, 95% confidence interval [CI: −0.11, 0.34] (Tables 3, 4). Findings for the decisiveness subscale indicated a significant time x group interaction, *p* = 0.001, *d* = 0.34, 95% confidence interval [CI: 0.14, 0.54] (Tables 3, 4). Findings for the emotional regulation subscale also indicated a significant time × group interaction, *p* = 0.04, *d* = 0.28, 95% confidence interval [CI: 0.03, 0.54] (Tables 3, 4).

The non-parametric Mann–Whitney *U*-test was used to compare between-group differences in social advising, insight, prosocial behavior, and tolerance for divergent values from baseline to 4 weeks. Results revealed no statistically significant differences between social advising scores in the intervention vs. control group at the end of study, *p* = 0.28, *d* = 0.04, 95% confidence interval [CI: −0.19, 0.27], insight scores, *p* = 0.65, *d* = −0.05, 95% confidence interval [CI: −0.3, 0.20], prosocial behavior scores, *p* = 0.95, *d* = −0.05, 95% confidence interval [CI: −0.27, 0.17], and tolerance for divergent values scores, *p* = 0.21, *d* = −0.33, 95% confidence interval [CI: −0.6, −0.06] (Table 3).

#### Psychological Well-Being

The normality and homogeneity assumptions for the personal growth and purpose in life subscales were not met. Moreover, ANOVA revealed significant differences at baseline scores on the purpose in life measure. Therefore, complete case analyses using GLM repeated measures with time x group interactions were only conducted on the subscales: positive relations with others, self-acceptance, environmental mastery and autonomy. Findings for the subscales indicated no significant time x group interaction on positive relations with others, *p* = 0.89, *d* = −0.01, 95% confidence interval [CI: −0.23, 0.2], self-acceptance, *p* = 0.54, *d* = 0.07, 95% confidence interval [CI: −0.16, 0.30], environmental mastery, *p* = 0.38, *d* = 0.11, 95% confidence interval [CI: −0.13, 0.34] and autonomy, *p* = 0.13, *d* = 0.19, 95% confidence interval [CI: −0.05, 0.42] (Tables 3, 4).

The non-parametric Mann–Whitney *U*-test was used to compare between-group differences in personal growth from baseline to 4 weeks. Results revealed no statistically significant differences between personal growth scores in the intervention vs. control group at the end of study, *p* = 0.73, *d* = 0.27, 95% confidence interval [CI: −0.0036, 0.55] (Table 3).

#### Subjective Well-Being

Table 5 presents the results for subjective well-being scores for each task in the app. Almost all participants (95.6%) from the complete case analysis had engaged in at least one mindfulness meditation throughout the study. Of those participants, 84.8% indicated they believed engaging in this practice helped improve their overall well-being. About half of the participants (53.6%) performed at least 1 cognitive restructuring task throughout the study. Of those participants, 70.3% believed engaging in this task helped improve their overall well-being. Journaling was used by 47.82% of the participants. 72.7% of those participants believed journaling had improved their overall subjective well-being. 65.2% of participants listened to either the music or nature sounds and 68.9% of those participants believed engaging in these tasks helped improve their overall well-being.

**Table 5:** Subjective-well-being by task.

	**Subjective well-being post-intervention**
**Out of those who engaged with each component**:	
Use of the mindfulness meditations has helped you improve your overall well-being.	
Yes	56 (84.8%)
No	10 (15.2%)
Use of the cognitive restructuring task has helped you improve your overall well-being.	
Yes	26 (70.3%)
No	11 (29.7%)
Use of the journaling task has helped you improve your overall well-being.	
Yes	24 (72.7%)
No	9 (27.3%)
Use of the music and/or nature sounds has helped you improve your overall well-being.	
Yes	31 (68.9%)
No	14 (31.1%)

## Discussion

Findings from the current study indicate individuals who engaged in Serene for 4 weeks demonstrated significant decreases in depression compared to the waitlist control condition. In addition to these decreases in clinical outcomes, significant increases in self-compassion (i.e., improvements in self-kindness, common humanity, and mindfulness and decreases in self-judgement, isolation and overidentification with one's negative emotions), emotional regulation and decisiveness were observed. Moderate between-group effect sizes were observed across all these measures except for emotional regulation (*d* = 0.28). A previous study by Mak et al. (2018) found apps that employ self-compassion and cognitive behavioral techniques can significantly reduce psychological distress. Moreover, the reported effects from the current study are in line with the efficacy of current mobile-based interventions that use similar techniques. For instance, a meta-analysis by Firth et al. (2017a) found a moderate effect size in reducing symptoms of depression through various mobile apps that employed cognitive behavioral and mindfulness meditation techniques (*g*^2^=0.38). Van Dam et al. (2014) also tested the change processes involved in predicting significant changes in depression through a mindfulness and self-compassion-based program and found reduced overidentification and self-judgement in conjunction with increased engagement in adaptive emotional regulation processes predicted changes in these clinical outcomes. Moreover, Diedrich et al. (2017) additionally found adaptive emotional regulation that involves an increased ability to tolerate one's negative emotions mediated the relationship between self-compassion and depression in individuals with unipolar depression. Serene can therefore help individuals cope with their depression in the current pandemic by reducing overidentification with one's negative emotions through self-compassion.

Although there were significant within-group decreases in anxiety in our current study, the results demonstrated no significant between-group differences in anxiety scores. A meta-analysis by Weisel et al. (2019) similarly found significant improvements in depression for mental health-based apps and no significant improvements over active controls for anxiety. Another meta-analysis by Firth et al. (2017b) however found a moderate effect size (*g*=0.45) on symptoms of anxiety for individuals with sub-clinical and clinical symptoms of anxiety who used apps that promoted overall mental well-being, some of which applied some CBT techniques. The reported differences may be due to the length and content of these interventions. Moreover, Canet-Juric et al. (2020) found individuals reported experiencing varying levels of anxiety over time during the pandemic. Individuals who quarantined alone were less likely to report experiencing anxiety levels over time. On the other hand, individuals who engaged in the consumption of COVID-related news were more likely to experience higher levels of anxiety. Therefore, more studies that examine the relationship between these factors and other potential influential factors (e.g., age differences) while engaging in Serene are needed. Engaging in Serene for a longer period (8–12 weeks) may also facilitate further significant reductions in anxiety symptoms.

Moreover, mean baseline scores in our current study were moderate on average for depression and anxiety and mild for stress. Participants also scored high on average on the psychological well-being measure. Therefore, it may be that participants in our current sample are already doing quite well. This warrants further studies with the Serene app in populations with significantly greater levels of anxiety, depression, and stress. Moreover, greater engagement with the app, and more specifically with the cognitive restructuring task may contribute to further significant effects across these measures. Shikatani et al. (2014) found both mindfulness and cognitive restructuring were essential in reducing cognitive distortions during post-event processing in individuals with social anxiety. Cuijpers et al. (2014) also found psychotherapeutic treatments that employ cognitive restructuring produced significant effects on anxiety symptoms. Since about only 50 percent of our participants engaged in this task, future studies may want to examine changes in anxiety in a larger sample in individuals who engaged in both mindfulness and cognitive restructuring. Moreover, 20 percent of individuals in the control group were engaging in mindfulness practices at the time of the study. Therefore, this may explain the equivalent decreases in the control group and the small between-group differences for stress and anxiety. Further studies may want to include individuals who have not engaged in any mindfulness practice for both conditions. Since the observed significant effects were evident after engaging with Serene for 4 weeks, the app may be providing these benefits at an early stage.

On average, individuals reported engaging with all aspects of the app (mindfulness practices, cognitive restructuring, and journaling) helped improve their overall subjective well-being. Most participants regularly engaged in mindfulness meditations every week. Cognitive restructuring was used at least once by almost 54% of the participants randomized to the intervention group. These results suggest that individuals who engage in Serene in as little as 4 weeks can benefit from its therapeutic effects. Mindfulness meditations included self-compassionate practices that were meant to not only help with attention regulation and awareness but also to address one's emotions through a semi-active role by viewing one's situation through a more holistic approach which allows for cognitive reframing to occur. By integrating these practices with a psychoeducational module, the hope was to facilitate a proactive approach to problem-solving one's challenges and to address one's emotional distress through a more compassionate approach. A meta-analysis by Aldao et al. (2010) revealed these forms of adaptive emotional regulation strategies (problem-solving, reappraisal, and acceptance), that are used throughout mindfulness and cognitive restructuring produce moderate to large effects on symptoms of anxiety and depression.

Prout et al. (2020) found the overall prevalence of anxiety and depressive symptoms has increased during pandemic, especially among younger females. Moreover, they found emotional regulation processes used by individuals were predictive of current distress experienced. Serene may offer an active emotional regulation strategy that can help improve COVID-related distress. Canet-Juric et al. (2020) also found individuals' levels of depression increased while their levels of positive affect decreased over time in areas where quarantine measures were in place. Specifically, this was more evident within the younger population. Moreover, Okruszek et al. (2020) and Killgore et al. (2020) found the social isolation measures implemented during the pandemic predicted increased loneliness, depressive and anxiety symptoms. Our results show Serene can reduce feelings of isolation and depressive symptoms. Due to the ongoing nature of these measures in place due to the pandemic, self-compassion practice using Serene may provide long-term benefits to protect individuals against these isolation-related effects from the pandemic which can exacerbate current psychological symptoms.

Lazarus and Folkman's (1984) transactional model on coping with stressors demonstrates the essential role of cognitive reappraisal in modulating stress appraisal. According to this model, appraisal of a stressor is related to one's interpretation of whether they deem a situation as threatening and whether they have the resources to deal with the situation (i.e., perceive it as controllable). Depending on these components, individuals will react in an adaptive way that contributes to increased levels of well-being, a sense of self-efficacy and positive affect or they will react in a way that will hinder their well-being, and promote greater negative affect (Garland et al., 2015). A recent study by Brose et al. (2020) found individuals who engaged in less adaptive appraisals of the pandemic i.e., perceiving greater threat and challenge in combination with a lower perceived sense of control and self-concept of one's own abilities, experienced increased negative affect, and less mindfulness in their daily life. Moreover, this form of stress appraisal was associated with increases in depressive symptoms and other negative psychological symptoms during the COVID-19-related lockdown in Germany.

Glück and Bluck's (2013) MORE Life Experience model regards wise individuals as those who understand life as being full of uncontrollable and unpredictable circumstances yet trust in their capabilities to engage in adaptive mechanisms in the face of these situations (Glück, 2021). Self-compassion practice may provide added benefits in combination with the cognitive restructuring task. Wong and Yeung (2017) demonstrated how self-compassion can significantly predict greater acceptance of a distressing situation through the recognition that one's current suffering is part of the human condition. Moreover, self-compassion practice results in a greater perception of controllability in stressful situations (i.e., perceiving that an individual has the capacity to deal with a stressor effectively; Chishima et al., 2018). Therefore, self-compassion can afford individuals the ability to engage with their stressors adaptively by not overidentifying with negative emotions associated with the event while recognizing that their experiences of suffering are shared with others. Engaging in the app may therefore allow individuals to have a more balanced perspective on their current feelings and situation. In addition, cognitive restructuring can then help one identify and modify unhelpful thoughts/beliefs that may be underlying these feelings and help one plan and develop steps to actively improve one's behavioral responses to these stressors which can subsequently contribute to improvements in depressive symptoms. In the face of the new resulting reality following the stressful events of the pandemic, wiser individuals may actively recruit these adaptive emotion regulation strategies. Through both compassion and wisdom, individuals can gain greater self-insight to care for their own welfare (Glück, 2021).

The Mindfulness-to-Meaning Theory by Garland et al. (2015) may also explain how self-compassion and mindfulness can provide an avenue for successful appraisal of a stressor through adaptive emotion regulation processes. Mindfulness practice helps shift the focus from one's automatic cognitive thought patterns to a non-judgmental metacognitive state of awareness. By shifting awareness to the present moment, individuals can view the situation holistically and thereby begin to notice other contextual information that can help them appraise a stressful situation in a more positive manner. For instance, individuals may notice available forms of support to them or they may shift their thought patterns from extreme cognitive distortions (e.g., all-or-nothing thinking or overgeneralizations) to more adaptive forms of thinking which can then help them formulate a more positive and meaningful appraisal of their situation (Garland et al., 2015). A greater sense of togetherness and comradery through self-compassion may then further facilitate this shift in cognitive processing to facilitate further improvements in depressive symptoms.

Our study revealed stable scores across both wisdom measures. However, there were significant between-group differences in emotional regulation and decisiveness in the San Diego-Wisdom subscales. These results are in line with the current conceptualization of wisdom by Ardelt (2003), Webster (2007), and Grossmann et al. (2016) as being a stable trait with some changeable context-influenced characteristics. For instance, individuals who engage in more wise reasoning, which can change based on the situation, are more likely to engage in positive reappraisal and reduced thought suppression while encountering a challenging situation. Grossmann et al. (2016) believes this form of wise reasoning predicts greater well-being, increased levels of positive affect and reduced negative affect. This also means that wisdom and well-being can be achieved through some of the strategies implemented in this app (i.e., self-compassion, cognitive restructuring, etc.).

Glück and Bluck's (2013)'s MORE Life Experience model also suggests wise individuals are able to reappraise challenging situations in a more positive manner and actively deal with them through the recruitment of certain psychological resources. For instance, increased emotional regulation, which can be acquired through self-compassion, can promote wiser reasoning which may help individuals deal with stressful situations more successfully (Glück, 2021). Moreover, wiser individuals are not only more aware of their feelings (both positive and negative) but they also learn to derive meaning from them and are able to recognize their importance in shaping their outcomes (Kunzmann, 2004). Similarly, self-compassion and mindfulness entail the recognition and understanding of one's current experiences and emotions as opposed to suppressing them or distracting oneself from remembering or dealing with them. Through self-compassion, individuals are aware of these negative emotions, yet they do not magnify their problems or allow them to dominate their core beliefs. Therefore, they can positively reappraise their current distressing situations through cognitive restructuring and learn and grow from them (Grossmann et al., 2016). Ardelt (2003) considers this form of reappraisal as a gateway to increased self-awareness and self-insight. In fact, one study by Sharma and Dewangan (2017) found engagement in an 18-week mindfulness and journaling-based intervention promotes increased levels of wisdom through the process of reappraisal. Therefore, individuals who engage with Serene can benefit from increased self-compassion levels that allow them to deal with stressors more effectively through adaptive emotional regulation and wise reasoning. This may ultimately contribute to improvements in pre-existing and new mental health challenges that have resulted from the pandemic. Given the wide availability of mental health apps, ensuring access to evidence-based interventions is vital. The results from this study suggest the need for methodological changes and incorporation of these critical ingredients in mobile-based mental health care.

### Limitations and Future Directions

Although most of the participants were female, the sample consisted of individuals from a wide range of educational backgrounds and employment statuses. The population mainly consisted of Canadian younger adults (Mean Age = 25.24), 25.5% of which met criteria for a variety of psychological clinical disorders. This aligns with the current percentage of adults who are diagnosed with a mental health disorder in other countries: 20.6% in the US and 19.8% for men and 32.4% for women among young adults in Norway (Gustavson et al., 2018; Substance Abuse Mental Health Services Administration, 2020). Therefore, our sample was representative of the composition of the general population in terms of mental health diagnoses. Additional research is needed to see whether these results translate for the male population and older adults in other geographical regions (e.g., United States, Europe, etc.).

Results for the stress variable demonstrated a small but non-statistically significant between-group difference in effect size between the intervention and control group using Cohen's *d* confidence interval. However, a statistically significant between-group difference was found for stress using the Mann-Whitney *U* test at the end of the study. Future studies may want to replicate this intervention in a larger sample over a longer period to conclude whether the observed effects translate to a significant between-group difference. Our results also demonstrated a moderate between-group difference in effect size between the intervention and control group using Cohen's *d* confidence interval for tolerance of divergent values. However, no statistically significant between-group difference was found for this subscale using the Mann–Whitney *U*-test. Moreover, mean change across the intervention group was small (−0.6). Future studies may want to explore possible extraneous variables that may explain these differences between these two groups. A larger sample size may also be able to detect these differences more accurately. Moreover, 8.7 to 18.8% of the sample reported experiencing pandemic-related significant changes over the 4 weeks that may have impacted their responses to these measures. Therefore, observed levels of stress, anxiety, and depression may have been influenced by these COVID-related factors. Overall, the app still appears to be effective regardless of these factors.

Moreover, more research on the efficacy of this intervention in a larger sample size with individuals with clinical diagnoses using structured diagnostic interviews is needed. Some research suggests men and women report experiencing different levels of positive and negative affect and that they utilize emotion regulation skills differently to some degree (McRae et al., 2008). Moreover, females report experiencing more depressive symptoms and rumination than males (Johnson and Whisman, 2013). Weststrate and Glück (2017) suggest wiser individuals who engage in adaptive forms of reflection (e.g., positive reframing) are more likely to have higher levels of well-being. In fact, Watkins and Roberts (2020) suggest tasks that involve continuous thought challenging or reappraisal combined with adaptive forms of coping may transform maladaptive ruminative behavior into an adaptive form of self-reflection. Therefore, more research on this appropriate utilization of the cognitive restructuring task (i.e., continuous rather than one-time) and whether it may contribute to reductions in ruminative behavior and subsequent depressive symptoms is needed. Grossmann et al. (2016) also suggests measuring wisdom through ecological momentary assessments to better capture one's levels of wisdom due to the dynamic nature of this construct that may change when one engages with different stressors. Therefore, future studies may incorporate this method to better understand whether other domains of wisdom can be improved by engaging with this app.

Although our sample was ethnically diverse, more research is also needed on investigating the efficacy of this intervention in a larger sample with ethnic minorities and individuals from economically disadvantaged backgrounds. Moreover, significant differences were found between the intervention and control group in regards to ethnicity (e.g., more individuals identified as South Asian in the control group). Further studies are needed to assess whether the results replicate in samples with similar ethnicity compositions. Future studies may also want to examine the efficacy of Serene against other current internet-delivered mental health interventions as opposed to a waitlist-control group to discern the most important therapeutic components that are needed in mental health apps that contribute to the reported outcomes. Further studies may want to employ serial mediation analyses to determine the role of some of the components of Serene that may be predicting these changes (e.g., increases in self-compassion, decreases in rumination or isolation).

Although the total wisdom score using the San Diego Wisdom scale had high internal reliability, the prosocial behavior subscale's reliability was questionable. Cronbach's alpha may be low for this subscale for a number of reasons e.g., using fewer items on a given scale often produces a lower reliability. Therefore, a more reliable scale may be needed to capture this construct. Moreover, non-significant changes in psychological well-being may be attributed to the low reliability scores for some of the subscale items. Subjective well-being was only measured through a binary Yes or No indicator as well. Therefore, well-established, and validated measures with higher internal reliability scores that better reflect well-being (e.g., Satisfaction with Life Scale; Diener et al., 1985) and emotion regulation (e.g., the Cognitive Emotion Regulation Questionnaire; Garnefski et al., 2001 or the Difficulties in Emotion Regulation scale; Gratz and Roemer, 2004), may be utilized to better capture the changes across these constructs. More research on the efficacy of this intervention long-term is also needed.

## Conclusion

Serene significantly improved outcomes for individuals experiencing depression. The app promotes increased self-compassion and emotion regulation which may facilitate these clinical improvements. Individuals may be able to transform their suffering during this challenging time through a sense of togetherness promoted through self-compassion. Together with the available adaptive problem-solving techniques, greater acceptance, positive reframing, and wise reasoning, individuals may then transform their maladaptive form of stress appraisal which may contribute to improvements in depression. Through these processes the app can promote resilience and recovery in the face of the additional distress created by the COVID-19 pandemic.

## Data Availability Statement

The raw data supporting the conclusions of this article will be made available by the authors, without undue reservation.

## Ethics Statement

The studies involving human participants were reviewed and approved by Social Sciences, Humanities and Education Research Ethics Board at the University of Toronto. The participants provided their written informed consent to participate in this study.

## Author Contributions

MA and AA contributed to the conceptualization of the study, data curation, formal analysis, investigation, methodology, project administration, resources used, visualization of material, and writing (original draft, review and editing). AA also developed the app (Serene). MM and NS also contributed to the conceptualization of the study, methodology, resources used and writing (review and editing). MF contributed to conceptualization of the study, methodology, writing (review and editing), and supervision of this project. All authors contributed to the article and approved the submitted version.

## Conflict of Interest

AA and MA are co-founders of the Serene App which will be commercialized. The remaining authors declare that the research was conducted in the absence of any commercial or financial relationships that could be construed as a potential conflict of interest.

## Footnotes

^1^https://www.randomizer.org/

^2^Hedge's *g* is also a measure of effect size. The difference between Hedge's *g* and Cohen's *d* is very small in large sample sizes > 20.
