# Supplementary material for: A Self-Compassion and Mindfulness-Based Cognitive Mobile Intervention (Serene) for Depression, Anxiety, and Stress: Promoting Adaptive Emotional Regulation and Wisdom
Source: Front Psychol. 2021 Mar 22;12:648087. doi: 10.3389/fpsyg.2021.648087 (PMC8019776; doi:10.3389/fpsyg.2021.648087)
Supplement: Supplementary file 1 [file Data_Sheet_1.DOCX]

Supplementary Material

# Psychoeducation Script

**Mindfulness** is a way of life. With mindfulness you will be able to increase your awareness to your own thoughts, feelings and body sensations. With kindness you will be invited to notice these things without really judging them or acting on them. For example, if you notice that you are starting to think about something that happened in the past, you would acknowledge these thoughts, and with kindness gently bring back your focus to the breath.

**Self-compassion** is one of many mindfulness-based practices. It also involves being in the present and being aware of our thoughts, feelings and body sensations but it also extends beyond that. Self-compassion involves being kind to oneself and recognizing that human suffering is a shared concept. We recognize the upsetting times we had as ones that might’ve happened to others as well. With self-compassion you will begin to view situations from this new perspective and not overidentify with your negative emotions.

So what’s the **evidence** on mindfulness and self-compassion practice? Well studies show that mindfulness and self-compassion decreases your stress levels, worry and anxiety, depression and rumination (or repetitively thinking about the past when you really don’t want to). It allows you to recognize what is helpful and unhelpful to you. Many individuals describe being able to have a new perspective on situations that were upsetting to them. This also translates to current and new situations they may face. Studies show that there is also significant improvements in one’s overall physical well-being and mental well-being and many people describe feeling greater life satisfaction. Practicing mindfulness will help improve your concentration on the current tasks that you’re doing. So by being present in the moment through mindfulness, you’ll be more mindful about the tasks that you do everyday. With mindfulness, you will be able to let the unhelpful thoughts flow through you.

Similar to the many meditations that you might’ve heard of or practiced before, mindfulness meditation incorporates focus on the breath or a sound. However, the goal is not to clear your mind or ignore incoming thoughts. Instead you are invited to accept them. And with practice you’ll be able to improve your mindfulness skills. So really the main emphasis here is on being kind to oneself throughout the whole process.

**So what happens when you start to practice mindfulness and self-compassion regularly?** Well when you begin to practice mindfulness, you’ll be able to strengthen your cognitive reframing muscle. What do we mean by cognitive reframing? Well cognitive reframing is the ability to look at a situation from a different perspective from your current one and this will help offer a new perspective on your current upsetting situation. So really mindfulness is a core foundational technique that you need to learn before you are able to engage in the next step of this program which is cognitive restructuring. Now cognitive restructuring is a way where we engage in some techniques to rebalance our thinking and address different upsetting situations in a healthy manner.

**Backdraft and other Upsetting emotions**

Backdraft is the emotional discomfort or uneasiness that may arise when we start to practice mindfulness and self-compassion. When we become more aware of our emotions and thoughts, we open the doorway to all kinds of emotions, some of which may discomforting. It can be confusing but in fact it’s a natural process that may occur and is part of this transformative process towards improving one’s well-being.

So what can you do about it? You may ask yourself what do I need right now to feel safe? Some things you can do is to allow these feelings to dissipate in the background while you continue your practices. You may also reduce engaging with some of these practices. Others might find it helpful to engage in mindfulness practice to regulate their attention and focus on the present instead of focusing on these other emotions. You may also practice validating yourself with a compassionate voice. You may say” It’s okay and label the emotion as one but not overidentify yourself with or judge it, and be kind yourself through this process” You may find an activity you enjoy or something that is soothing for you such as going out with a friend or having a nice warm cup of coffee.

You may also choose to reach out to a family member, friend, a family doctor, therapist or a helpline if upsetting emotions arise. Some practices may elicit some emotions in people depending on their past experiences. Many people who experienced trauma in the past for instance may find focusing on something other than the breath more helpful. For example, you may use the nature sounds/music meditations as your focus. You may engage in mindful writing i.e. focusing on a writing project, or you may practice mindfulness while walking or eating. The goal is to redirect the attention on an activity while still being present with the activity. This takes away the focus from our internal experiences which may cause some upsetting emotions. Moreover, many of our meditations also include a waterfall sound in the background which one may focus on instead.

So in essence, this depends on how you perceive the uneasiness and upsetting feelings and these are a few things you can do when these emotions come up.

**We briefly discussed cognitive restructuring in the previous module on mindfulness. Let’s look at this technique in more detail.**

**Cognitive Restructuring** involves the identification and modification of unhelpful thoughts or beliefs underlying our feelings. By using this tool, you will be able to make different choices and develop and apply new behaviours and strategies. Combining it with mindfulness and self-compassion practices can help you develop awareness of the processes that maintain these habitual patterns of unhelpful thoughts and beliefs. For example, mindfulness can help you pay attention to both the physical and cognitive or mental signs of these feelings you experience. For example, you might experience tension in the body which may symbolize feelings of worry or anxiety. You might also start to repetitively think about moments in the past which may symbolize feelings of sadness or depression. Through mindfulness practice, you’ll be able to develop greater awareness of these signs. Developing greater awareness of these signs will help us see the situation that is associated with these thoughts and feelings from a more balanced perspective which fosters acceptance of these situations. And with acceptance you will be able to reframe these unhelpful thoughts or feelings that may come up. You’ll also learn some techniques for coping with these upsetting feelings or thoughts. Finally, you will learn how to deal with the different upsetting situations that you want to address in a healthy manner through comprehensive action plans that you will develop.

**Situation:** So following mindfulness practice, you are invited to use cognitive restructuring to assess a recent or past situation that was upsetting to you. You may also identify a situation that you anticipate may be upsetting in the future and analyze how you might want to deal with it in a healthy manner.

**Feelings:**  After you identify the situation, you may either try and recall some of the feelings you observed during mindfulness practice or when you experienced the situation. We may not be able to identify some of the feelings we experienced easily. For instance, some people describe anxiety as a feeling of tension in one’s shoulders or whole body. Some people may see they feel like there are butterflies in their stomach. People who experience sadness or depression may describe it as a cloudy feeling in their head or feeling like they are experiencing a headache. You may also experience more than one of these feelings at the same time.

**Thoughts:**  When you think of a situation, along with the different feelings you might experience, you might also have different kinds of thoughts related to the situation. We often listen to our own thoughts and our gut instinctual feelings and base our decisions on them. However, many of our thoughts may be helpful or unhelpful. Unhelpful thoughts are the ones that may prevent us from moving forward. For instance, when we continue to think about an incident in the past, it may prevent us from moving forward which perpetuates an unhealthy lifestyle. Although our thoughts are often linked to our feelings, we need to unpack them and determine which are supported by real evidence (or facts) and which aren’t. Some examples of common unhelpful styles of thinking include: all-or-nothing and self-blame thinking. For example one might say: I can not do anything in my life. I’m a failure. Others may magnify their problems and minimize their achievements. Thinking that we “should” and “must” do things is another common unhelpful style of thinking. For example you might think: I must get an A+ or I should be able to do this since everyone else likely can!)

With cognitive restructuring, you will balance real evidence for and against this type of thinking. You will critically analyze the evidence by looking at the quality of the evidence rather than the quantity. Now let’s examine one example of an unhelpful thought and analyze the evidence for it. Sam thinks that her friend Sarah does not care her in a meaningful way. Some evidence against this thought may be the fact that Sarah is always there for Sam when she’s sick and brings her work material when she misses work. She also responds to her late night calls when Sam is worried about something she had to deal with that was stressful. Some evidence for this thought may be: that often Sam and Sarah generally have superficial conversations rather than meaningful ones. Examining the evidence for and against the thought that Sam and Sarah don’t have a meaningful and caring relationship, we may see there is some minimal support to the thought but overall the thought is not supported by the evidence.

**Rebalancing our thinking:**  If the thought is not supported by any evidence or is only partially supported by the evidence we may want to examine the thought from a different perspective. Based on the previous example we may want to rebalance the thought to a more appropriate one. This can be achieved through a number of ways. We may practice mindfulness which will help us become more aware of the other hidden helpful qualities of Sarah that we may have not realized. This will allow us to look at this thought from a new perspective and come up with a more balanced thought. So a more balanced thought for this scenario may be that. Maybe Sarah really cares about me when it really matters. And that we just need to open up more to each other so we can continuously support each other. Therefore we have rebalanced our thinking to match the evidence and have also developed a plan to improve our relationship with Sarah.

**Action plan:** If the evidence does not support the thought or only partially supports it, along with rebalancing our thinking we may want to create an action plan to cope with any upsetting feelings that may arise due to the situation. What’s an example of an action plan? Well we may want to practice some mindfulness techniques for instance if we experience any anxiety to ground ourselves during these moments. We may want to practice the loving-kindness meditation if we start feeling that someone doesn’t care about us like in our example which will help us not only rebalance that thinking but it will also promote engaging in compassion and understanding towards ourselves.

Action plans are also mainly used if the evidence supports the thought in some way. In an action plan we may also develop some techniques to cope with any upsetting feelings or thoughts that come up. These are also called self-care techniques where the focus is all about YOU. In addition to that we may create a plan to address the problem head-on. In our previous example, we may have decided to have a conversation with Sarah about our concerns and we may decide to initiate a meaningful conversation once a week with Sarah as our first step. We may also decide to practice this program for example which is another way of addressing the problem head on. It may also be helpful to incorporate some resources in our action plan. Some individuals may find it helpful to have important contacts handy. We may reach out for support from a close friend, family member, our family doctors or a local helpline in case any upsetting emotions arise).

There are different ways to learn to cope with different situations. Some of them are more helpful than others. We’ve previously brought up some examples of these coping mechanisms. Let’s look at 2 different ways of coping in a healthy way. The first way is called **emotion-focused coping** and it may include acceptance and the ability to positively reframe (which can be achieved through mindfulness and self-compassion practices that are used in this program). There are other types of emotion-focused coping. For example we may use humour. We may reach out for emotional comfort and support from our loved ones or we may engage in religious or spiritual practice.

The second type of coping is called **problem-focused coping**. Problem-focused coping is what we termed as addressing the problem head-on. We may another type of support that is more instrumental rather than one that is for emotional comfort. SO we may seek out a therapist, we may use evidence-based techniques such as cognitive restructuring like what we’re doing right now, or we may engage in planning. Previously we mentioned that Sarah had planned a few steps to initiate meaningful conversations with her friend. This is one example of planning. The next step would be to implement and work on this plan. There are also other forms of active coping which is also a problem-focused coping mechanism. For example we may exercise which would not only improve our physical health but also provide us with other benefits such as boosting our mental health as well!)

Often the most effective coping strategies are healthy and are enjoyable in some way. Try and think about similar activities you’ve done in the past that are both healthy and enjoyable for you so you can incorporate them into your plan. These are the kinds of coping strategies that you are more likely to use!

**Journaling**

Journaling can be used as a tool for emotional release. We can write down our thoughts and feelings about things that bother us or we can write about positive experiences we may have. We may also track our progress in the cognitive restructuring task. Tracking our progress helps us identify things that we’ve tried that were helpful for us and things that were not when we’re developing an action plan. It can also be used to generally track changes in our mood. For instance we may notice changes in our mood when we include something in our daily routine such as exercise or mindfulness practice.

Mindful writing is also one exercise that combines journaling and mindfulness. Instead of focusing on the breath as we learned from the previous mini-course on mindfulness meditations, we instead shift our focus to the writing project. A writing project might be a story that you’re working on, or you might be planning for a future event. Whatever the project is, the goal is to kindly redirect your focus on the project if your mind starts to wander.

All of us have moments in our life when we question ourselves and feel insecure, guilty or shameful, we wonder if we’re good enough if we’ve done enough in situations. We may try using an exercise called the self-compassion letter. In this journaling-based exercise, you may think of similar moments. Then you may think of someone who supports you, a family member, a friend or an imaginary friend who is compassionate towards you, treats you well, and supports you. Imagine if this person knows all your strengths and all your limits. They recognize all your faults and all that’s positive about you, They are able to forgive you, accept you for who you are. From that perspective, imagine if that friend was writing a letter to you, what would they tell you if they’re coming from a place that’s filled with love, compassion, kindness and forgiveness? What would they do to encourage you feel these things towards yourself, how would they encourage you to change in a kind and compassionate way? You may also instead think of moments when you are providing that great support and compassion to someone close to you. When you start to feel that compassion for them, gently redirect that compassion towards yourself.

After writing this letter you may read it back to yourself in moments when you need support and compassion in moments that are upsetting to you. Feel the words and allow them to flow through you to fill yourself with compassion. You may revisit this exercise and add more to it. You may not be able to write much or find any words to say and that’s okay. It’s a process and you will slowly become more aware of your emotions and thoughts. Remember to be kind to yourself throughout this process.
